# 

**Published:** 1891-10

**Authors:** 


### LITERARY.

### "THE CENTURY'S" LIFE OF COLUMBUS.

The *Century Magazine* will celebrate the 400th anniversary of the discovery of America, by publishing a Life of Columbus, written specially for that magazine, by Emilio Castellar, the famous Spanish orator, statesman and author. The work is written in Spanish, and will be carefully translated. Señor Castellar, whose interest in and admiration for America are well known, has made a careful study of the new historical material bearing upon the subject, and it is said that his papers will be very richly illustrated. Other articles dealing with the discovery of America, are in course of preparation for the same magazine.

---

GEN. H. V. BOYNTON, the well-known Washington correspondent, has written an article on "The Press and Public Men," for the October *Century*.

---

The several authors of the following essays will please accept our thanks:

THREE PAPERS.—"Quinine in Typhoid Fever;" "The Influence of the Maternal Mind Over the Unborn Child;" and "Maternal Nursing *versus* Artificial Feeding;" read before the Montgomery Co., Indiana, Medical Society, by J. W. Stanghan, M. D.

ILLUSTRATED CASES OF CONGENITAL CLUB FOOT. By Prof. Augustus Wilson, M. D., Professor of General and Orthopædic Surgery, etc., Philadelphia.

TRACHOMA AND ITS TREATMENT. By W. Cheathmam, M. D., University of Louisville, Ky.

---

AS A NERVE TONIC, USE HORSFORD'S ACID PHOSPHATE.—Dr. S. L. Williams, Clarence, Iowa, says of it: "For a nerve tonic, I think it is the best I have ever used, and can recommend it most confidently."

